# The LP-ESP^®^ lumbar disc prosthesis with 6 degrees of freedom: development and 7 years of clinical experience

**DOI:** 10.1007/s00590-012-1166-x

**Published:** 2013-01-11

**Authors:** Jean-Yves Lazennec, Alain Aaron, Adrien Brusson, Jean-Patrick Rakover, Marc-Antoine Rousseau

**Affiliations:** 1 Department of Orthopedic and Trauma Surgery, La Pitié-Salpétrière Hospital, 47-83 boulevard de l’Hôpital, 75013 Paris, France; 2 Biomechanics Lab, Arts et Métiers Paritech, 151 boulevard de l’Hôpital, 75013 Paris, France; 3 FH Orthopedics, 68990 Heimsbrunn, France; 4 Department of Anatomy, Université Pierre et Marie Curie Paris 6, 105 boulevard de l’Hôpital, 75013 Paris, France; 5 Clinique du Pré, 72000 Le Mans, France

**Keywords:** Disc arthroplasty, Disc replacement biomechanics, Motion preservation, Disc degeneration, Low back pain, Spinal alignment

## Abstract

The viscoelastic lumbar disk prosthesis-elastic spine pad (LP-ESP^®^) is an innovative one-piece deformable but cohesive interbody spacer providing 6 full degrees of freedom about the 3 axes, including shock absorption. A 20-year research program has demonstrated that this concept provides mechanical properties very close to those of a natural disk. Improvements in technology have made it possible to solve the problem of the bond between the elastic component and the titanium endplates and to obtain an excellent biostability. The prosthesis geometry allows limited rotation and translation with resistance to motion (elastic return property) aimed at avoiding overload of the posterior facets. The rotation center can vary freely during motion. It thus differs substantially from current prostheses, which are 2- or 3-piece devices involving 1 or 2 bearing surfaces and providing 3 or 5 degrees of freedom. This design and the adhesion-molding technology differentiate the LP-ESP prosthesis from other mono-elastomeric prostheses, for which the constraints of shearing during rotations or movement are absorbed at the endplate interface. Seven years after the first implantation, we can document in a solid and detailed fashion the course of clinical outcomes and the radiological postural and kinematic behavior of this prosthesis.

## Introduction

Because of its impairment of patients’ personal, social, and professional lives, degenerative disk disease has become an important public health problem with multiple dimensions. The current therapeutic strategy remains controversial and is also a medical and surgical challenge. Conservative treatment, mostly based on physical therapy, constitutes the first-line approach, but persistent symptomatic disease may be treated surgically in selected patients [1–3]. Lack of pain relief, stiffening of the lumbar spine, nonunion, sagittal balance misalignment, bone graft donor site morbidity, and, last but not least, adjacent segment disease are the pitfalls of intervertebral fusion that led to the idea of total disk replacement (TDR) [4–7]. Since 1966 and Fernström’s first TDR implantation [8], many designs and concepts have been proposed [9–18]. The devices are usually articulated implants, and their mobility depends on the designs of the bearing surfaces. Ball-and-socket two-piece prostheses have 3 degrees of freedom in every rotation around a single fixed center of rotation. Three-piece devices allow additional translation components, providing 5 degrees of freedom. Articulated TDRs have demonstrated their clinical utility in several patient series. Specifically, the non-inferiority of TDR versus fusion is now generally accepted [13–15, 19]. However, in vitro testing of the two types of implants reveals that both designs have biomechanical advantages and limitations.

Because the healthy human intervertebral disk has a deformable elastic structure with 6 degrees of freedom, elastomeric one-piece intervertebral prostheses might be the most physiological implant for mimicking physiological levels of shock absorption and flexural stiffness. Designing such a device is challenging, especially when we remember the Acroflex^®^ prostheses: The elastic rubber failed so rapidly in vivo that only 28 were implanted in all [20, 21].

The LP-ESP^®^ (lumbar disk prosthesis-elastic spine pad) was developed over a 20-year period. Improvements in technology have made it possible to solve the problem of the bond between the elastic component and the titanium endplates. After successful in vitro and in vivo evaluation, the LP-ESP has been authorized for clinical use in Europe since 2005. The goal of this paper is to present its innovative concept and the clinical results and radiological outcomes over its 7 years of use.

## Implant design

**Fig. 1:**
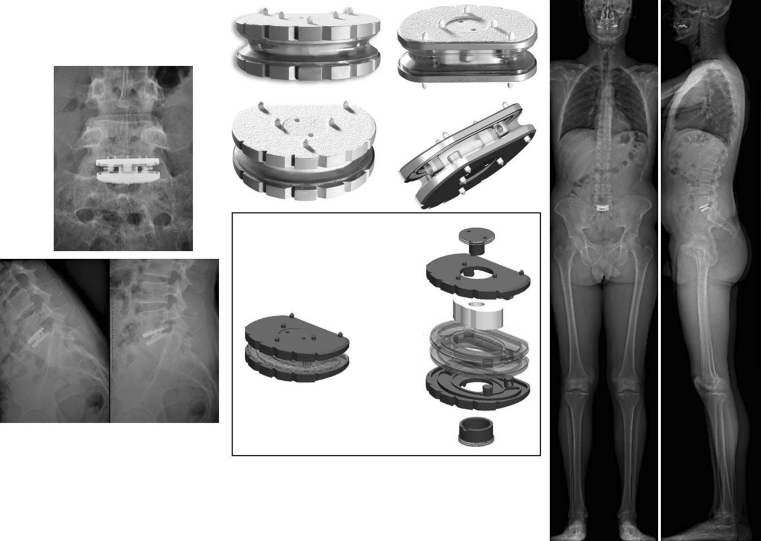
The design of the LP-ESP^®^ prosthesis is based on the principle of the silent block bush

**Fig. 2:**
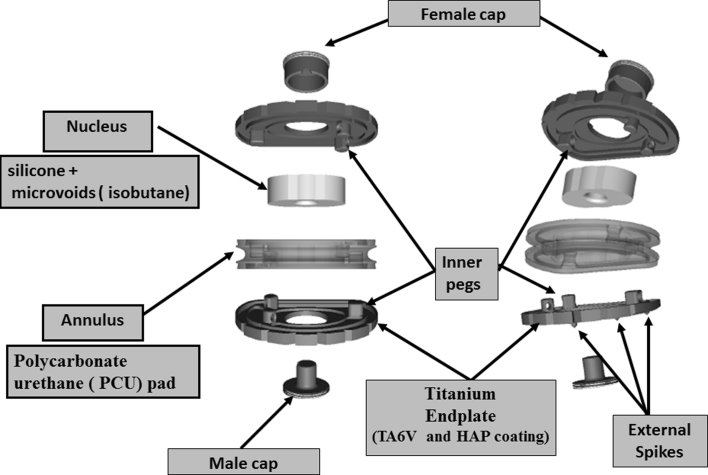
The LP-ESP^®^ is a one-piece deformable implant including a central core made of silicone gel with microvoids and surrounded by polycarbonate urethane (PCU) securely fixed to titanium endplates

The design of the LP-ESP^®^ prosthesis is based on the principle of the silent block bush (Fig. 1). The LP-ESP^®^ is a one-piece deformable implant including a central core made of silicone gel with microvoids and surrounded by polycarbonate urethane (PCU) securely fixed to titanium endplates (Fig. 2). The endplates have five anchoring pegs to provide primary fixation and are covered by a textured T 40 titanium layer (60 μm thick) and hydroxyapatite to improve bone ingrowth.

Depending on the size, the titanium endplates differ in thickness and angulation. The prostheses are available in two thicknesses (10 and 12 mm), each with 3 angles of lordosis (7°, 9°, and 11°). Regardless of the model, however, the mechanically active cushion and the mechanical properties of the prosthesis are the same: The differences in thickness of the lordotic angle do not affect the prosthesis’s mobility or its cushioning, even shock-absorbing, effect.

**Fig. 3:**
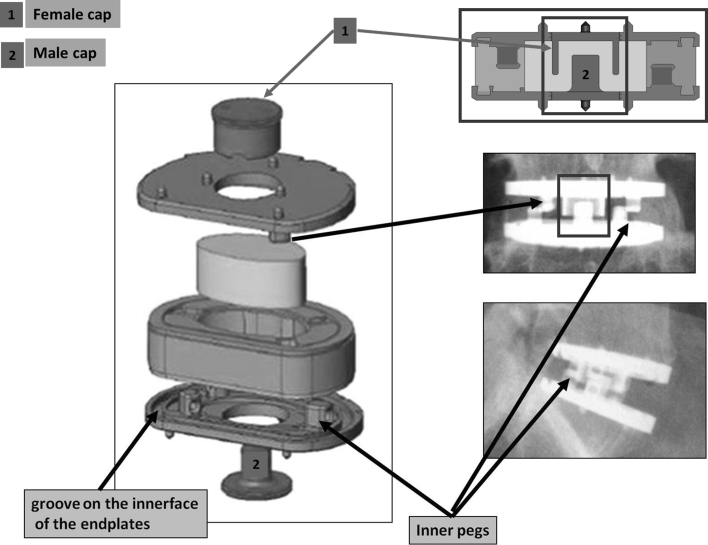
The peripheral cushion (the annulus) is securely fixed to the titanium alloy endplates by adhesion-molding technology. This attachment is reinforced by a peripheral groove without the addition of glue. The PCU annulus is stabilized by supplementary pegs located on the internal surface of both metal endplates. The cushioning and compressing effects are obtained on the one hand by the contactless interlocking of the *male* and *female* caps and, on the other hand, by crushing the annulus between the two metal plates

Accordingly, the peripheral cushion (that is, the annulus) is securely fixed to the titanium alloy endplates by adhesion-molding technology. This attachment is reinforced by a peripheral groove without the addition of glue. This process of fixation avoids fluid infiltration and the risk of fatigue fractures of the interface, despite the very different mechanical properties of the polymer and the metal endplates. The PCU annulus is stabilized by supplementary pegs located on the internal surface of both metal endplates. The geometry and position of the pegs, between the peripheral groove and the central area of the endplates, were planned to control rotational mobility (Fig. 3). The polymer molding was designed to prevent all direct contact between the upper and lower pegs.

The core or nucleus is composed of a compressible silicone structure containing isobutane microbubbles. This core is injected after the annulus surrounding it has been molded. Two titanium caps allow the core to be contained at the moment of the injection. These two pieces are firmly secured to the titanium plates: They also play a mechanical role by their contactless fit, because they contribute to limiting shearing during anteroposterior and mediolateral translation. The cushioning and compressing effects are obtained on the one hand by the contactless interlocking of the male and female caps and, on the other hand, by crushing the annulus between the two metal plates. The same components limit the shearing effect when the endplates are inclined to the horizontal.

On the whole, in the LP-ESP, the constraints of the interface between the PCU cushion and its titanium seating are reduced. These are principally constraints of compression:

- between the exterior of the male cap and the interior of the female cap for translations;
- between the pegs for the rotation;
- between the titanium endplates for flexion.

The principle of the LP-ESP^®^ makes it possible to reproduce the anisotropy of the healthy disk, and the design allows modification of the return torque (without modifying the other parameters of the prosthesis. For example:

- bringing the pegs closer together increases stiffness in rotation without modifying either translation or compression;
- diminishing the clearance between the male and female caps increases the stiffness of translation without modifying either rotation or compression;
- modifying the ratio between the small and large diameters of the elliptic form of the cushion changes the ratios of the return torques between flexion/extension and lateral flexion without modifying stiffness in compression or rotation.

## Design stages

After an initial patent application in 1994 by R. ROY CAMILLE, different avenues of research were explored, with the scientific expertise of the CEA (Commissariat à l’Energie Atomique, Fontenay aux Roses, France) and the industrial expertise of FH Industry for further R & D (Heimsbrunn, France). The preliminary stages involved optimizing the choice of PCU, the development of the attachment of the annulus to the metal endplates without chemical adhesives, the definition of the pegs and caps, and the implementation of reliable techniques for polymer molding and injection. Biocompatibility tests were performed by BIOMATECH, a subsidiary of NAMSA (Northwood, Ohio, USA).

**Fig. 4:**
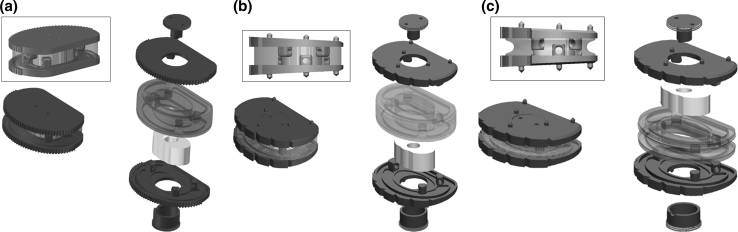
**a** First generation of lumbar ESP^®^ (2004): endplates without lordosis. **b** Second generation of lumbar ESP^®^ (2005): The shape of the endplates provides lordosis. **c** Last generation of lumbar ESP^®^ (2006): anterior recess in the PCU annulus to reduce stiffness during compression

Human implantation began in 2004 with the first generation of LP-ESP^®^ implants, which used endplates without lordosis (40 implantations, all complying with the Huriet Act, which defines French ethical requirements). A second generation of implants with lordotic endplates (7°, 9°, and 11°) was introduced in 2005—LP-ESP 1. A final change was made to the PCU annulus in 2006: Its periphery is no longer rectilinear but was recessed somewhat during the molding process. This change did not modify the attachment of the cushion of the LP-ESP 2^®^ prosthesis but made it possible to reduce its stiffness during compression by 30 % without changing its characteristics for flexion/extension, lateral incline, or rotation (Fig. 4a–c). This ESP prosthesis received CE marking in 2005, making it the first elastomeric lumbar prosthesis to be validated and authorized for marketing.

## Mechanical properties

The “silent block bush” design of the LP-ESP^®^ prosthesis avoids the disadvantage of centers of rotation that are fixed or controlled by the implant design, as observed in disk prostheses based on an articulated design. In addition, in each direction solicited, the prosthesis offers resistance that increases with the amplitude of the movement. In this sense, the LP-ESP^®^ cannot be compared to first-generation implants. It meets the mechanical criterion of 6 degrees of freedom and provides a cushioning effect while restoring elastic recovery properties. Its mechanical properties are close to those reported in the literature for the normal disk (see Table 1).

**Table 1:** Comparison of the mechanical properties of the LP-ESP 2^®^ prosthesis with those of the natural disk

Pure moments applied in increments up to a maximum value of 10 N/m	References	Level	Natural disk	LP-ESP 2^®^ prosthesis
Flexion–extension	Panjabi [22]	L4/L5	6°	6°
		L5/S1	4°	
	Campana [24]	L1/L2	4°	
		L4/L5	7°	
	Yamamoto [23]	L1/L2	5°	
		L4/L5	7.5°	
Lateral flexion	Panjabi [22]	L4/L5	4°	2.5°
		L5/S1	2°	
	Campana [24]	L1/L2	4.1°	
		L4/L5	6.1°	
	Yamamoto [23]	L1/L2	5°	
		L4/L5	5.7°	
Torsion	Panjabi [22]	L4/L5	2°	2°
		L5/S1	1°	
	Campana [24]	L1/L2	2.4°	
		L4/L5	3.4°	
	Yamamoto [23]	L1/L2	2.3°	
		L4/L5	2.2°	
Axial compression	Gardner-Morse [25]		2,420 N/mm	2,300 N/mm
Variable according to the loading speed, values retained for 0.1 m/s	Virgin [26]		3,000 N/mm	
	Kemper [27]		1,835 N/mm	
	Bouzakis [28]		1,700 N/mm	
Elastic recovery			Yes	Yes

## Biomechanical assessment

The originality of the concept of the ESP^®^ prosthesis led to innovative and intense testing of various sorts.

### Structural tests

#### Creep tests

After continuous compression to 1,250 kN for 2,928 h (122 days), the height loss was 0.2 mm. In the 8 h following load removal, the residual height loss was 0.1 mm.

#### Influence of the pegs included in the PCU annulus to control rotations

Tests were performed for combined compression and rotation: The pegs included in the PCU annulus absorb approximately 50 % of the torque.

#### Assessment of the cohesion of the prosthetic cushion and the metal endplates

**Fig. 5:**
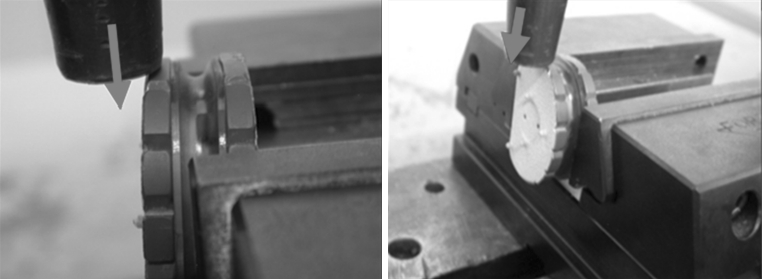
Assessment of the cohesion of the prosthetic cushion and the metal endplates: The tests were performed for mediolateral and anteroposterior exertion applied to one of the metal endplates, with the other plate attached to the test machine

The tests were performed for anteroposterior and mediolateral exertion applied to one of the metal endplates, with the other plate attached to the test machine (Fig. 5). For implants 12 and 10 mm thick, respectively, a force of 450 and 800 N was required to obtain a gap of 1 mm between the 2 endplates in the anteroposterior direction and 550 and 600 N in the mediolateral direction.

#### Maximum compression tests

These tests were inspired by the experimental protocol of Virgin [26], who stated that a natural healthy disk is irreversibly injured by a load of 3–11 kN. After application of a force of 4,800 N (100 h) and then 9,200 N (64 h), we did not observe irreversible destruction of the implants. Compression tests and then compression–shearing at an angle of 45° were performed on the same samples to obtain successive compressions of 2, 3, and 6 mm. These tests show the implant’s excellent tolerance of these compression–shearing mechanisms.

#### Tests to validate the final stage of coating on the exterior side of the metal plates

**Fig. 6:**
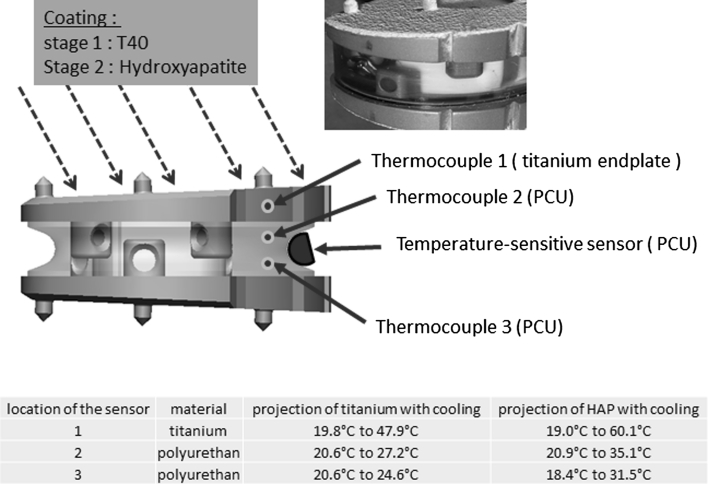
Thermosensitive indicators confirm that the temperature of the polyurethane remained below 65 °C. The appearance of the polymer remains unchanged

Adding a further final coating of porous titanium and spraying hydroxyapatite on the implant in its permanent form causes its temperature to rise. During the coating process, the disc is cooled by compressed air so that the ambient temperature remains stable at 21 °C. Tests were performed to verify the absence of any effect from this rise on the mechanically active cushion in view of the known risk of PCU deterioration at 120 °C. These tests demonstrated that the temperature did not reach a level of risk to the PCU (Fig. 6).

### Fatigue and wear tests

**Fig. 7:**
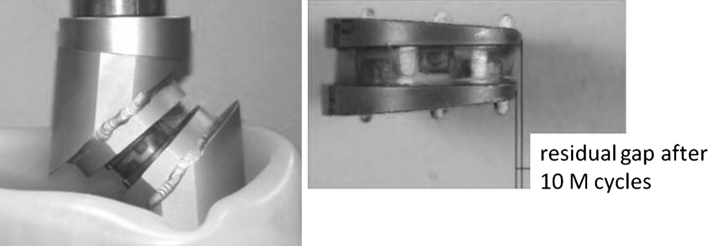
Wear tests were conducted in a 3-axis motion simulator (frequency = 4 Hz, loads of 135–1,350 N). The inclination of the prosthesis was 45° to reproduce the sagittal orientation of the disk in functional situations. The gap between the metal plates was measured after each series of 10 million cycles

Wear tests were conducted in a 3-axis motion simulator according to the following protocol (Fig. 7):

- Series of 10 million cycles of flexion, extension, and lateral tilting
- Frequency = 4 Hz
- Loads of 135–1,350 N
- Inclination of the prosthesis at 45° to reproduce the sagittal orientation of the disk in functional situations
- In a demineralized water bath at 37 °C

**Fig. 8:**
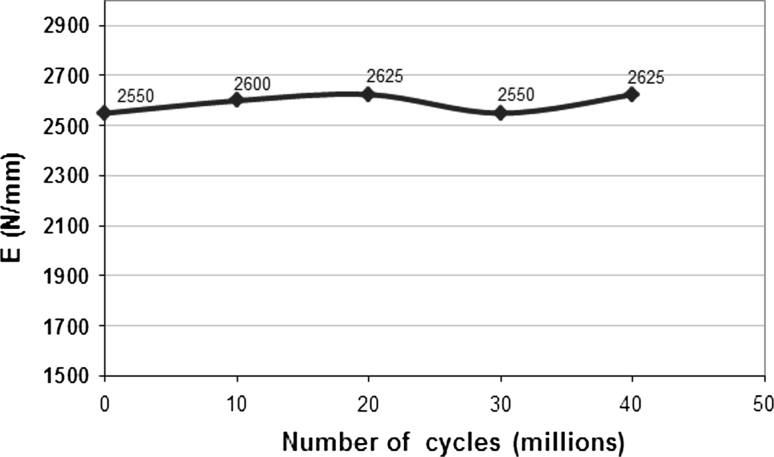
Stiffness in compression

**Fig. 9:**
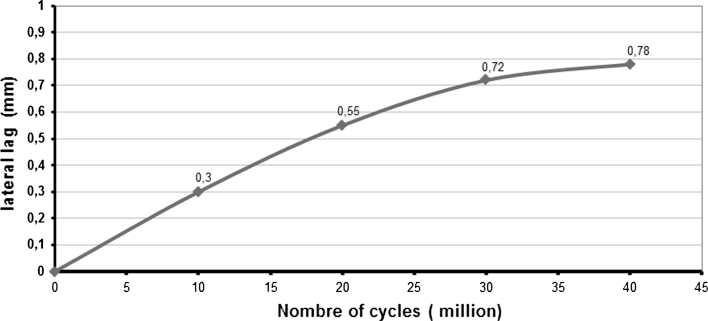
Evolution of the lag of the endplates plates

Tests have even been extended to 40 million cycles without any observation of signs of mechanical failure. No loss of cohesion was seen and stiffness remained stable (Fig. 8). The residual gap between the metal endplates was 0.55 mm after 20 million cycles and 0.78 mm after 40 million cycles (Fig. 9). Loss of mass after 20 million cycles was less than 0.5 % (very low absorption of saline solution and slight degradation of the endplates coating).

### Biostability tests

This test was conducted according to the requirements of IS0 standard 10993-13/biological evaluation of medical devices, Part 13: Identification and quantification of the decay products of polymer-based medical devices.

The biostability of the implant was assessed by analysis of the particles collected during the filtration of the demineralized water bath, after a wear test of 10 million cycles under a load of 1,350 N. This study used a scanning electron microscope (SEM LEO1455VP), equipped with an energy-selective spectrometer (EDS OXFORD). No particles from the component materials of the prosthesis were found.

The tests looking for salted out or released matter showed the emission of <1 mg/kg methylene diphenyl 4-4 diisocyanate and of 64.9 mg/kg of 4-4 methylene diamine. These results are consistent with the data in the literature [29].

### PCU aging test

The specific PCU (Bionate 80A) used for the LP-ESP prosthesis is not oxidized during storage (DSNM Biomedical The Netherlands, according to master file MAF844). Kurz demonstrated that 5 years of shelf aging has little effect on the mechanical properties of the PCU and concludes that the bionate 80A material has greater oxidative stability than ultra-high molecular weight polyethylene following gamma irradiation in air and exposure to a severe oxidative challenge [30]. Tests were performed after artificial aging in water at 80 °C followed by 10 million compression cycles at loads ranging from 150 to 1,250 N. In the absence of published standards in the literature, the temperature was determined in comparison with that recommended for aging plastics, including UHMWPE (ASTM standard F 2003: Accelerated aging of ultra-high molecular weight polyethylene after gamma irradiation in air), and the axial load was that recommended by ISO standard 18192 (Intervertebral spinal disk prostheses—Part 1: Loading and displacement parameters for wear testing and corresponding environmental conditions for test) for wear tests. It was not observed significant changes in the stiffness of the implants tested.

**Fig. 10:**
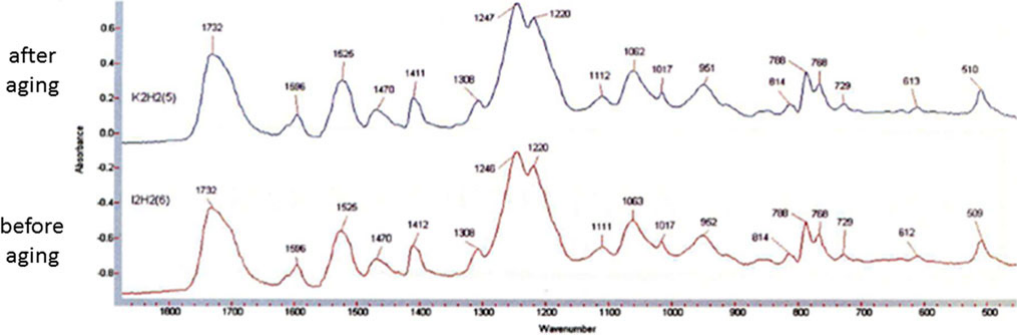
Comparison of infrared spectra after/before PCU aging: No change in the spectra was observed

No modification of the Fournier transform infrared spectrum or any modification of the mean molecular weight (ASTM standard D 5296) was observed (Fig. 10). The chemical composition and organization of the atomic bonds, therefore, remained identical because oxidation or natural cross-linkage would have modified the atomic organization and thus the spectrum. These results are consistent with the literature [29].

## Biocompatibility tests

All the materials were studied separately and in their final assembly, meeting the specifications for biocompatibility tests described in ISO standard 10993 (Biological Evaluation of Medical Devices). Tests were performed by Biomatech (Chasse-sur-Rhône, France).

- Cytotoxicity test according to ISO standard 10993-5
- Sensitization test according to ISO standard 10993-10
- Test of irritation or intradermal reaction according to ISO standard 10993-10; acute systemic toxicity according to ISO standard 10993-11 Chromosomal genotoxicity (heart test and chromosomal anomalies according to ISO standard 10993-3)
- The implants also meet the criteria of the FDA’s subacute sensitization test (following FDA—Guidelines for Toxicity Tests Chapter IV).

## Clinical results

### Evaluation process

As of today, more than 2000 LP-ESP II prostheses have been implanted. No complication related to the materials has been reported. The clinical experience can be illustrated by the analysis of a prospective series of 120 patients who are representative of the current use of the ESP implant (2 surgeons JYL and JPR) with a complete 2-year follow-up. There were 73 women and 47 men in this group. The average age was 42 (27–60). The average body mass index (BMI) was 24.2 kg/m² (18–33). The implantation was single level in 89 % of cases; 134 ESP prostheses were analyzed.

The functional results were measured using:

- the Oswestry Disability Index (ODI): a 15 % improvement of the ODI at 2-year follow-up was considered as a good result according to previously published criteria [31, 32].
- the SF-36, with distinguishing the physical component (PCS) and the mental component (MCS),
- the GHQ 28 (General Health Questionnaire) that was used for psychological assessment [33],
- the visual analogic scale (VAS) regarding back pain [34].

**Fig. 11:**
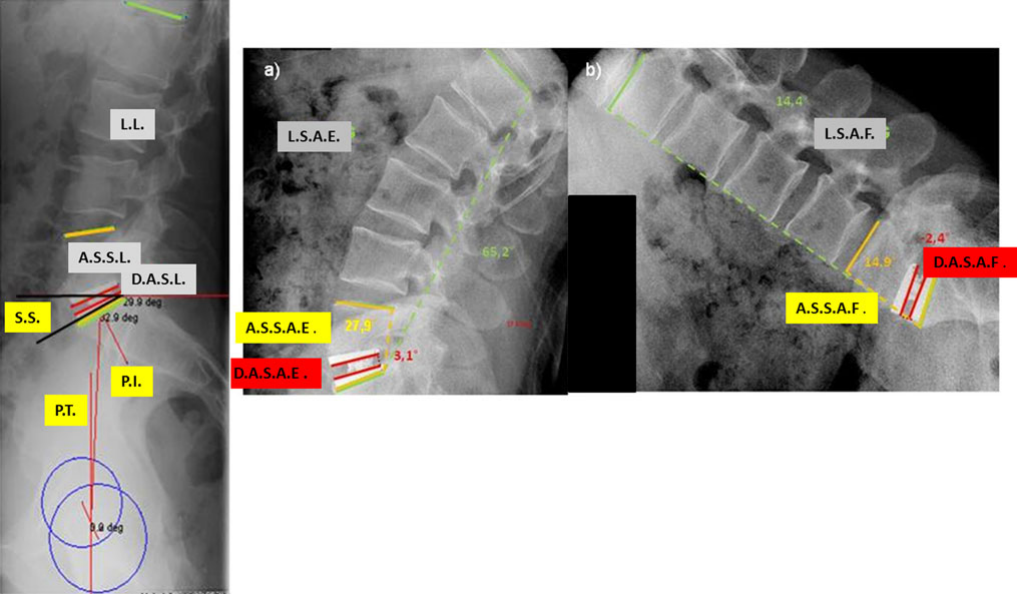
Measurement of lumbopelvic parameters and calculation of range of motion on flexion/extension X-rays. *L.L.* lumbar lordosis, *A.S.S.L.* adjacent segment segmental lordosis, *D.A.S.L.* disc arthroplasty segmental lordosis, *S.S.* sacral slope, *P.I.* pelvic incidence, *P.T.* pelvic tilt, *L.S.A.E.* lumbar sagittal angle for extension, *L.S.A.F.* lumbar sagittal angle for flexion, *A.S.S.A.E.* adjacent segment sagittal angle for extension, *A.S.S.A.F.* adjacent segment sagittal angle for flexion, *D.A.S.A.E.* disc arthroplasty sagittal angle for extension, *D.A.S.A.F.* disc arthroplasty sagittal angle for flexion: D.A.S.A.F. Intervertebral flexion–extension angle is calculated according to Wong [40, 41]: (D.A.S.A.E)–(D.A.S.A.F.) for the arthroplasty level. (A.S.S.A.E.)–(A.S.S.A.F.) for the adjacent level

Clinical data and X-rays were collected at the preoperative time and at 3, 6, 12, 24, and 36 months postop. Paired t-test were used for comparing the outcomes at each time point post-op to their pre-operative values. The level of significance was set for *p* < 0.05. The radiological images were digitized using the dedicated scan Vidar™ Twain 32 (Vidar Corporation) and analyzed using the Dicomeasure software (Viewtec, Maisons-Alfort, France) [35]. The analysis was performed by a single observer (AB) who was independent from the selection of patients and from the surgical procedure. On lateral views in standing position were measured the sacral slope (SS), the pelvic tilt (PT), and the segmental lordosis (SL). Mobility of the prosthesis, the upper adjacent level, and L1S1 segments were measured on flexion/extension X-ray (Fig. 11). One recall that the inferior limit for the criteria of mobility is 3° for some authors [36] and 5° for others [37–39]. The difficulty in obtaining strict lateral views of the spine limited the radiological analysis of kinematic to 67 patients (74 prostheses). In accordance to the work of Wong et al. [40, 41] and Lee et al. [42], the interpretation of the mobility of the prosthetic level has been considered in reference to the age and the global mobility of the L1S1 segment.

### Clinical results

The mean operative time was 92 min (standard deviation, SD: 49 min). The mean blood loss was 73 cc (SD: 162 cc).We did not observe device-related specific complications. The clinical results are reported in Tables 2 and 3. All clinical outcomes significantly improved at every time points when compared to the pre-operative status. The series demonstrated an improvement of 18.7 % for GHQ, 26.6 % for SF 36 PCS, and 16.4 % for SF 36 MCS. At the final follow-up, an ODI improvement >5 points is observed in 76 % of the cases. ODI improvement of 15 is 85 % at 24 months and 90 % at 36 months. ODI improvement of 25 is 85 % at 24 and 36 months.

**Table 2:** Description of the different clinical evaluations performed

Mean ± SD	PREOP	3 months	6 months	12 months	24 months
VAS	6.6 ± 1.7	3.7 ± 1.9	3.4 ± 2.1	3.5 ± 2.3	3.4 ± 2.4
ODI (%)	47.6 ± 14.6	30.3 ± 17.6	24.5 ± 17.6	21.8 ± 16.3	20.6 ± 17.3
GHQ 28	64.2 ± 15.6	52.5 ± 14.7	52.7 ± 15.8	52.2 ± 15.4	50.6 ± 15.4
SF 36 PCS (%)	32.4 ± 34.8	48.4 ± 39	51.9 ± 39.3	55.6 ± 39.8	59 ± 39.2
SF 36 MCS (%)	42.3 ± 34.0	50.8 ± 34.6	52.8 ± 35.6	53 ± 36.3	58.7 ± 34.6

**Table 3:** Improvement of the Oswestry Disability Index (ODI) in reference to the preoperative status (in % of the population)

	3 months	6 months	12 months	24 months
ODI improvement of 15 %	72 %	82 %	85 %	85 %
ODI improvement of 25 %	59 %	75 %	82 %	83 %

In the series, 89 % of patients had a good or excellent result at 3 months, 88 % at 6 and 12 months, and 93 % at 24 months.

### Radiological outcome

Tables 4 and 5 summarize the changes in the radiological parameters of sagittal balance and the variations of range of motion (ROM) over time.

**Table 4:** Radiological parameters of sagittal balance in standing position over time (mean ± SD)

	PREOP	3 months	6 months	12 months	24 months
Pelvic incidence (PI)	54.8 ± 8				
Sacral slope (SS)	40.4 ± 7.2	41 ± 6.6	40.6 ± 6.8	41.2 ± 6.2	41.4 ± 6.8
Pelvic tilt (PT)	14.3 ± 7.3	11.8 ± 7	12.3 ± 6.2	12.4 ± 6.7	12 ± 7
Lumbar lordosis	55.8 ± 10	58.5 ± 12.5	59.2 ± 11.3	59.4 ± 13.5	58.3 ± 13.1

**Table 5:** Range of motion (ROM) of the prosthesis, of the upper adjacent disk, and of the lumbar spine L1S1 over time. (mean ± SD)

	3 months	6 months	12 months	24 months
ROM of the instrumented level	4.1 ± 2.4	4.7 ± 2.8	6.0 ± 3.4	5.3 ± 3.2
ROM of the upper adjacent level	4.9 ± 3.2	6.0 ± 4.7	7.9 ± 5.2	6.2 ± 4.1
ROM of the lumbar spine	24.3 ± 14.	27.9 ± 17.9	34.6 ± 16.3	27.5 ± 17.2

More than 3° of ROM is observed in 66 % of the prosthesis, and more than 5° in 60 % of the implants. According to the previous publications of Wong et al. [40, 41], we correlated the ROM observed at the disc replacement level with the physiological mobility in relation to the age of the patients and the global lumbar mobility. In the age group corresponding to our population, the physiological L1S1 global mobility is 30°, mean L4L5 ROM is 4.5°, and mean L5S1 ROM is 2.5°. In the present series, L1S1 global mobility was 28°, mean L4L5 ROM 4.6°, and mean L5S1 ROM 2.7°.

## Discussion

The viscoelastic LP-ESP^®^ prosthesis achieves 6 degrees of freedom including vertical translation; it provides a cushion and may allow shock absorption. A 20-year research program has demonstrated that this concept provides mechanical properties very close to those of a natural disk. The geometry of the implant allows limited rotation and translation with resistance to motion aimed at avoiding overload of the posterior facet joints. The center of rotation can vary freely during motion.

It thus differs substantially from other current prostheses, which are 2- or 3-piece devices involving 1 or 2 bearing surfaces and providing 3 or 5 degrees of freedom, with no or very little resistance, and no elastic return. This design and the adhesion-molding technology differentiate the LP-ESP prosthesis from other mono-elastomeric prostheses, for which the constraints of shearing during rotations or movement are absorbed at the plastic/titanium interface because of the molding technology used in their design. The attachment is obtained by the penetration of the polymer through small holes in the endplates. This process creates multiple interfaces and potential fatigue lesions of the anchoring mechanism due to inhomogeneous loading during flexion–extension, lateral inclination, and rotation. Thus, in these designs, the plastic mono-block cushion secured to the titanium plates flows into the space between them during compression, creating an area of friction and wear.

In addition, the biostability of the implant was demonstrated: No particles from the component materials of the prosthesis were found after a wear test of 10 million cycles under a load of 1,350 N. These experimental data should be considered in relation to previously reported results from Nechtow et al. [55] of wear rates of 16.59 ± 0.96 mg/million cycles for ProDisc-L and 19.35 ± 1.16 mg/million cycles for Charite, and from Grupp et al. [56] of wear rates ranging from 0.14 ± 0.06 mg/million cycles to 2.7 ± 0.3 mg/million cycles for Active L. Moreover, the size and morphology of the UHMWPE particulates observed in these studies are similar to those described in total hip and knee replacements [57], the osteolytic potential of which is well known.

Seven years after the first implantation, we can document in a solid and detailed fashion the course of clinical outcomes and the radiological postural and kinematic behavior of this prosthesis. We acknowledge that more studies with more patients and more follow-up would be useful in the future to assess long-term reliability. Nonetheless, the series reported here describe the outcomes that might be expected by surgeons and patients over the first 7 years. These encouraging results are basically comparable to the clinical results reported with the Prodisc II^®^ [43], the SB Charite^®^, [44], as reported in Table 6.

**Table 6:** Mobility described in the literature of implants restoring more than 5° of segmental mobility

Author	Years	Follow-up	Type of prothesis	L3–L4 ROM	L4–L5 ROM	L5–S1 ROM	Global ROM
Gioia [45]	2007	2	Charité III	8	10.3	8	8.7
Bertagnoli [46]	2005	2	Prodisc	–	–	–	6.5
Guyer [47]	2009	2	Charité	–	6.5	6	6.3
		5		–	6	6.3	6.2
David [48]	1993	1	Charité	2	9.4	6.4	5.9
Siepe [49]	2007	1	Charité III	–	7.2	5.9	6.5
Zigler [50]	2012	5	Prodisc	–	–	–	7.7
Delamarter [51]	2011	2	Prodisc-L	7.8	6.2		7.0
The present series	2012	2	LP-ESP	–	8.2	7.6	7.9

Sagittal parameters showed no major imbalance in spinal posture. These results are consistent with those reported in the literature with articulated prostheses by Chung et al. [52] and Tournier et al. [36, 53]. We note that publications do not appear to report significant sagittal misalignments after prosthetic implantation, whereas lumbar fusion may deleteriously alter the sagittal balance of the spine, including a decrease in the SS and lumbar lordosis [6, 7]. The increased segmental lordosis might be related to the lordotic shape of the prosthesis but also probably to the fact that arthroplasty, in contrast to fusion, allows the lumbar spine to find a new balance spontaneously. It has not yet been demonstrated, however, that this self-adaptation of the sagittal balance protects against adjacent-level degeneration. Unlike arthrodesis, the preservation or restoration of some mobility with a total disk replacement aims at limiting overload of the adjacent levels.

The optimal ROM after TDR for limiting adjacent segmental disease has not yet been established. Huang et al. [54] reported a series of 42 Prodisc I^®^ implantations with 8.7 years of follow-up, and 24 % of the junctional levels showed radiological signs of degeneration. In their study, the mean ROM of the disk prostheses adjacent to junctional disease was significantly lower than the mean ROM of the prostheses adjacent to a radiologically normal disk, that is, 1.6° versus 4.7°. Prevalence of junctional degeneration was 0 % among patients with ROM of 5° or more and 35 % among those with less than 5°. The authors did not conclude that 5° was the trigger value for avoiding adjacent degeneration, as 65 % of patients with less than 5° did not develop adjacent segmental degeneration. In our series, the LP-ESP^®^ device provides mobility levels similar to those with articulated prostheses such as Prodisc, which vary according to the series from 3.8° to 13.2° [52, 54]. Because of the normal variation of spinal motion of subjects in different age ranges, interpretation of ROM of disc prostheses should be careful and mainly about the minimum and maximum values for flexion–extension. As the population concerned by lumbar disc replacement cannot be compared to a group of young healthy subjects regarding the motion profile, we suggest that the reference for ROM should take into account the global lumbar mobility as well as the age of the patients to analyze more accurately the functional outcome of implants. Taking into account these parameters, the average values for flexion–extension observed in this series are in agreement with previously published reference data [40–42].

We recognize that assessing spinal kinematics with static X-rays in flexion and extension is subject to bias, given the same-day variations due to inconsistent effort during flexion/extension [58]. Nonetheless, flexion/extension X-rays are easily available and cause less irradiation than continuous motion analysis with in vivo fluoroscopy.

## Conclusion

The concept of the LP-ESP^®^ prosthesis is different from that of the articulated devices currently used in the lumbar spine. This innovative one-piece deformable but cohesive interbody spacer provides 6 full degrees of freedom about the 3 axes. In addition, the elastic return property provides a potential protection for the posterior facet joints.

The 7-year clinical experience provides encouraging clinical results about pain, function, kinematic behavior, and radiological sagittal balance after implantation of the LP-ESP^®^.
